# Associations Between Parental Expectations and Competitive State Anxiety in Adolescent Tennis Players: Mediation by Basic Psychological Needs

**DOI:** 10.3390/children12121714

**Published:** 2025-12-18

**Authors:** Zhaoyuan Chen, Lu Peng

**Affiliations:** Department of Human Sports, Kangwon National Univesity, Samcheok 25913, Gangwon-do, Republic of Korea; chenzhaoyuan727@gmail.com

**Keywords:** parental expectations, competitive state anxiety, basic psychological needs, competence, adolescent athletes

## Abstract

**Highlights:**

**What are the main findings?**

- Excessive parental expectations significantly increase competitive state anxiety and negatively impact the satisfaction of basic psychological needs in adolescent tennis players.
- Competence satisfaction specifically mediates the relationship between parental expectations and anxiety, whereas autonomy and relatedness do not play a significant mediating role.

**What are the implications of the main findings?**

- This study identifies a “dual-pathway” mechanism where parental pressure acts both as a direct external stressor and as a psychological burden that erodes perceived competence.
- Interventions aimed at reducing pre-competitive distress should prioritize protecting athletes’ self-efficacy and managing parental pressure, particularly in individual sports contexts.

**Abstract:**

Background/Objectives: In youth competitive sports, excessive parental expectations are frequently identified as critical external stressors that can impair athlete well-being. Grounded in Self-Determination Theory (SDT), this study aimed to investigate specific psychological needs that mediate the relationship between parental expectations and competitive state anxiety, particularly within the context of adolescent tennis players. Methods: A sample of 420 adolescent tennis players participated in this study. Participants completed self-report questionnaires, including the Parental Expectations Questionnaire for parental expectations, the Chinese version of the Basic Psychological Needs Scale for basic psychological needs (autonomy, competence, and relatedness), and the Chinese version of the Competitive State Anxiety Inventory-2 for competitive state anxiety. Data were analyzed using regression models and mediation analysis to test the hypothesized relationships. Results: Parental expectations significantly and positively predicted competitive state anxiety (B = 0.111, *p* < 0.01). Furthermore, high parental expectations were negatively associated with the satisfaction of basic psychological needs, specifically demonstrating a significant negative prediction of competence satisfaction (β = −0.18, *p* = 0.005). Mediation analysis revealed that the need for competence significantly and partially mediated the relationship between parental expectations and anxiety (B = 0.019, *p* < 0.001), whereas the indirect effects of autonomy and relatedness were non-significant. Conclusions: Findings indicate that parental expectations function both as a direct stressor and a psychological burden that specifically undermines the athlete’s sense of competence. These results highlight that in individual competitive sports, the erosion of self-efficacy is the primary psychological conduit linking parental pressure to pre-competitive distress.

## 1. Introduction

Concomitant with the professionalization and intensification of youth sports, the psychological burden placed on young athletes has become increasingly salient [1]. Pre-competitive state anxiety, defined as a negative emotional state characterized by nervousness, worry, and apprehension, is widely recognized as a critical risk factor in athletic performance. Recent empirical evidence highlights that elevated anxiety levels significantly impair physiological readiness [2] and disrupt cognitive decision-making processes [3]. Elevated anxiety levels have been linked not only to increased susceptibility to injury but also to burnout and eventual dropout from sport [4]. According to the Multidimensional Anxiety Theory, this anxiety manifests as cognitive concerns regarding performance failure and somatic physiological arousal [5]. In individual sports like tennis, where athletes face isolated performance accountability without the buffering effect of teammates, susceptibility to such anxiety is significantly exacerbated [6]. Consequently, identifying the social and psychological antecedents of competitive anxiety is imperative for safeguarding athlete welfare.

Among the various social agents in a young athlete’s environment, parents serve as a primary source of influence [7]. While parental support is integral to athletic development, parental involvement can manifest as a “double-edged sword,” [8]. Specifically, parental expectations, which refer to the standards and achievement goals parents set for their children, often function as a significant external stressor when they exceed the athlete’s coping resources [9]. Research grounded in the Transactional Theory of Stress suggests that high parental expectations are frequently appraised by adolescents as social evaluative threats, triggering an immediate anxiety response [10,11]. Empirical studies, such as 2010 in youth competitive tennis [12] and 2022 in youth basketball [13], have consistently demonstrated that perceived pressure from parents is a robust predictor of fear of failure and performance distress.

However, the mechanism by which external parental expectations translate into internal anxiety remains to be fully elucidated. To unpack this psychological pathway, this study adopts Self-Determination Theory (SDT) as a guiding framework [14]. SDT posits that optimal psychological functioning depends on the satisfaction of three basic psychological needs: autonomy, competence, and relatedness. In the context of youth sports, high parental expectations, particularly when perceived as controlling or contingent upon success, can create a rigid environment that directly thwarts these needs [15]. Instead of fostering intrinsic motivation, such controlling environments compromise the athlete’s sense of autonomy by making participation feel coerced, while unrealistically high standards erode their sense of competence [16,17].

Furthermore, the frustration of these basic needs is a direct precursor to maladaptive psychological outcomes. According to SDT, when athletes feel ill-equipped to meet situational demands (competence frustration) or lack a sense of ownership over their actions (autonomy frustration), their adaptive regulation collapses [18], leading to heightened psychological distress [19]. This link is particularly salient in racket sports like tennis, where the high technical precision and individual responsibility require robust psychological stability. Empirical evidence in racket sports indicates that threats to competence and autonomy are potent predictors of pre-competitive anxiety, as the athlete perceives a lack of control over performance outcomes [20,21]. Specifically, a deficit in perceived competence is closely linked to the cognitive component of anxiety in tennis players [22].

Integrating these relationships, we propose a mediation model. We argue that parental expectations operate not only as a distal environmental stressor but also as a proximal psychological burden that transmits its effect through the erosion of the athlete’s internal resources. While some direct effects of pressure may persist, SDT suggests that the deleterious impact of controlling social environments on well-being is largely explained by the thwarting of basic needs [13,23]. Specifically, in an achievement context like tennis, the pressure to meet parental standards may primarily attack the athlete’s sense of capability and volition, which in turn fuels anxiety [24,25].

**Figure 1:**
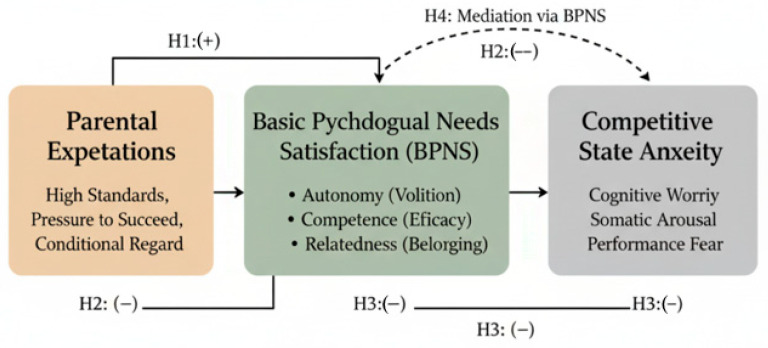
The Research Hypothesis Model.

By testing these hypotheses using Structural Equation Modeling (SEM) with a sample of adolescent tennis players, this study aims to clarify the “dual pathways” through which parental expectations influence athlete well-being, providing specific insights into the distinct roles of competence, autonomy, and relatedness. Figure 1 depicts the hypothesized structural model grounded in Self-Determination Theory. Consequently, the primary objective of this study is to examine the “dual pathways” through which parental expectations influence athlete well-being, focusing on the mediating role of basic psychological needs. Based on the theoretical framework and literature discussed above, we propose a research model and posit the following hypotheses:

**H1.**  *Parental expectations will positively predict competitive state anxiety in adolescent tennis players.*

**H2.**  *Parental expectations will negatively predict the satisfaction of basic psychological needs (autonomy, competence, and relatedness).*

**H3.**  *The satisfaction of basic psychological needs will negatively predict competitive state anxiety.*

**H4.**  *Basic psychological needs satisfaction will mediate the relationship between parental expectations and competitive state anxiety.*

## 2. Materials and Methods

### 2.1. Participants

The study sample was selected using a cluster random sampling method, where specific regional tennis training teams were defined as the cluster units. The target population comprised junior high school-level tennis team members across the Guangxi region in China. A total of 420 questionnaires were distributed. After excluding invalid responses (e.g., incomplete data or regular patterning), 412 valid questionnaires were retained, yielding an effective response rate of 98.09%.

The final sample consisted of 412 adolescent athletes, comprising 213 males (51.7%) and 199 females (48.3%). Regarding the age distribution, to ensure non-overlapping categories, participants were grouped as follows: 108 were under 12 years old (26.2%), 160 were aged 12–13 years (38.8%), 84 were aged 14–15 years (20.4%), and 60 were aged 16 years or older (14.6%). All participants were active members of regional representative teams competing at the provincial junior level. They reported an average training experience of approximately 3.5 years, with a weekly training volume ranging from 12 to 18 h.

### 2.2. Measures

#### 2.2.1. Parental Expectations

Perceived parental expectations were assessed using the Parental Expectations Questionnaire revised by Cheng (2010) [26]. This scale measures the adolescents’ subjective perception of their parents’ expectations across five: Academic Performance (4 items), Future Development (4 items), Conduct (3 items), Interpersonal Relations (6 items), and Physical/Mental Quality (7 items), totaling 24 items. Responses were rated on a 5-point Likert scale ranging from 1 (Completely non-conforming) to 5 (Completely conforming), with higher scores indicating higher levels of perceived parental expectations.

**Figure 2:**
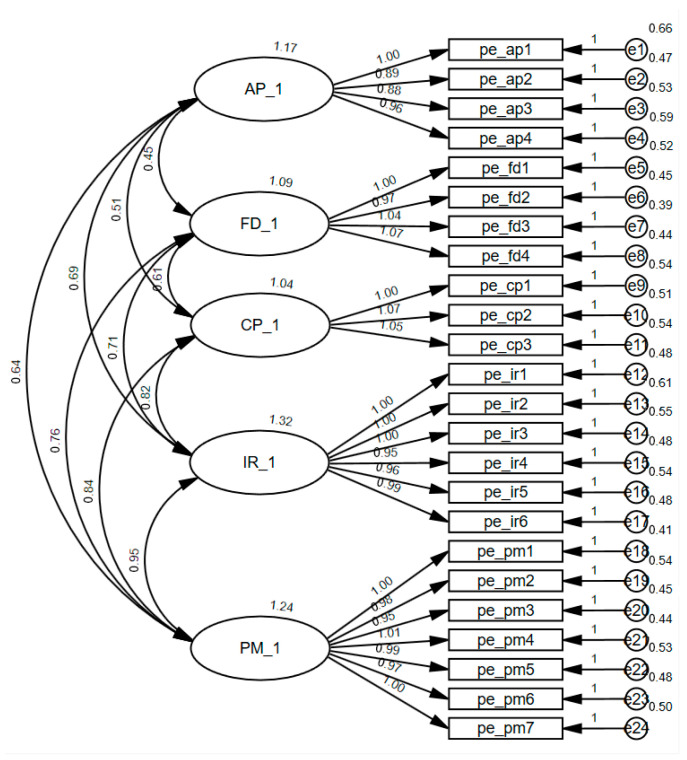
The CFA Model.

To confirm the construct validity of the Parental Expectations Scale, a Confirmatory Factor Analysis (CFA) was conducted using AMOS (see Figure 2). The five-factor measurement model demonstrated an excellent fit to the data ($\chi^{2}=236.68$, df=242, p=0.584). Comparative fit indices indicated a high degree of model compatibility, with CFI=1.00, NFI=0.97, GFI=0.953, and RFI=0.966. Furthermore, the Root Mean Square Error of Approximation (RMSEA) was $<0.001$, falling well below the recommended threshold of 0.05. These results substantiate the multidimensional structure and construct validity of the scale for the current sample. Model fit was evaluated using standard indices with the following acceptable thresholds adopted: $\chi^{2}/df$ < 3, CFI > 0.90, TLI > 0.90, and RMSEA < 0.08 [27].

#### 2.2.2. Basic Psychological Needs Satisfaction (BPNS)

The Chinese version of the Basic Psychological Needs Scale, originally developed by Deci et al. (2001) and adapted for the Chinese context [28], was used to assess the satisfaction of Autonomy, Competence, and Relatedness in sport and life contexts. The scale consists of 19 items across three dimensions: Competence (6 items), Autonomy (5 items), and Relatedness (8 items). Participants responded on a 7-point Likert scale ranging from 1 (Completely non-conforming) to 7 (Very conforming). The KMO value for this scale was 0.949, supporting its structural validity. The scale demonstrated strong internal consistency, with Cronbach’s α coefficients of 0.937 for competence, 0.931 for Autonomy, and 0.962 for Relatedness. The total scale reliability was 0.949.

#### 2.2.3. Competitive State Anxiety (CSAI-2)

Pre-competitive anxiety was measured using the Chinese version of the Competitive State Anxiety Inventory-2 (CSAI-2), revised by Zhou (2000) based on Martens et al. (1990) [5,29]. The inventory comprises 27 items assessing three subscales: Cognitive State Anxiety (9 items), Somatic State Anxiety (9 items), and State Self-Confidence (9 items). Items were rated on a 4-point intensity scale ranging from 1 (Not at all) to 4 (Very much so). The KMO value was 0.933, indicating the data were suitable for factor analysis. The internal consistency was satisfactory across all dimensions: Cognitive Anxiety (α = 0.860), Somatic Anxiety (α = 0.865), and Self-Confidence (α = 0.822). The overall scale reliability was 0.933.

The reliability and validity indices for all measures are summarized in Table 1.

### 2.3. Procedure

The study protocol was approved by the local ethics committee and the relevant sports administration authorities. Data collection was conducted at the training bases of the participating teams. To minimize recall bias and ensure the ecological validity of the state anxiety measures, questionnaires were administered approximately one hour prior to a scheduled provincial-level competition or an intensive training match (defined as full-court intra-team ranking matches with officiated scoring). The questionnaire administration was strictly supervised by trained research assistants to ensure protocol adherence and answer any queries. Research assistants explained the purpose of the study and emphasized the anonymity and confidentiality of the responses. Informed consent was obtained from all participants and their guardians. The average completion time was approximately 15 min.

### 2.4. Data Analysis

Data were processed and analyzed using SPSS 27.0 and AMOS 27.0 (IBMCorp., Armonk, NY, USA). First, descriptive statistics (means, standard deviations) were calculated. Second, the reliability (Cronbach’s α) and validity (KMO and Bartlett’s Test of Sphericity) of the measurement tools were examined. Prior to model testing, assumptions of normality, linearity, and multicollinearity were verified. Finally, Structural Equation Modeling (SEM) was conducted using AMOS 27.0 to test the hypothesized relationships between parental expectations, basic psychological needs, and competitive state anxiety. Model fit was evaluated using standard indices with the following acceptable thresholds adopted: $\chi^{2}/df$ < 3, CFI > 0.90, TLI > 0.90, and RMSEA < 0.08 [27].

## 3. Results

### 3.1. Common Method Variance Test

Given that data for all study variables were collected via self-report questionnaires from the same participants at a single time point, there is a potential risk of Common Method Variance (CMV). To address this, procedural remedies (e.g., ensuring anonymity and reverse-coding items) were implemented during data collection. Regarding statistical verification, although the CFA marker variable approach is often preferred, the current study design did not include an a priori marker variable theoretically unrelated to the substantive variables. Consequently, statistical verification was conducted using Harman’s single-factor test [30]. An exploratory factor analysis (EFA) was performed on all measurement items using an unrotated factor solution. The results revealed that while multiple factors with eigenvalues greater than 1 were extracted, the first factor accounted for only 21.11% of the total variance. This is well below the critical threshold of 40% suggested by Podsakoff et al. [31], indicating that CMV is not a pervasive issue in the current dataset and does not compromise the validity of the results.

### 3.2. Descriptive Statistics and Correlation Analysis

Table 2 presents the means, standard deviations, and Pearson correlation coefficients for all variables.

First, significant positive correlations were observed among the five dimensions of Parental Expectations (r = 0.348 to 0.705, *p* < 0.01).

Second, Parental Expectations were negatively correlated with the satisfaction of Basic Psychological Needs. Specifically, Physical/Mental Quality expectations showed significant negative associations with competence, autonomy, and relatedness needs (r = −0.181 to −0.239, *p* < 0.05 or 0.01).

Third, the three subscales of BPNS (Competence, Autonomy, and Relatedness) demonstrated moderate to strong positive inter-correlations (r = 0.435 to 0.547, *p* < 0.01).

Finally, regarding Competitive State Anxiety, the satisfaction of all three basic needs was significantly negatively correlated with Cognitive and Somatic Anxiety (r = −0.133 to −0.246, *p* < 0.05 or 0.01) and positively correlated with State Self-Confidence (r = 0.190 to 0.278, *p* < 0.01). Additionally, a strong positive correlation was found between Cognitive and Somatic Anxiety (r = 0.862, *p* < 0.01).

### 3.3. Structural Equation Modeling and Mediation Analysis

**Figure 3:**
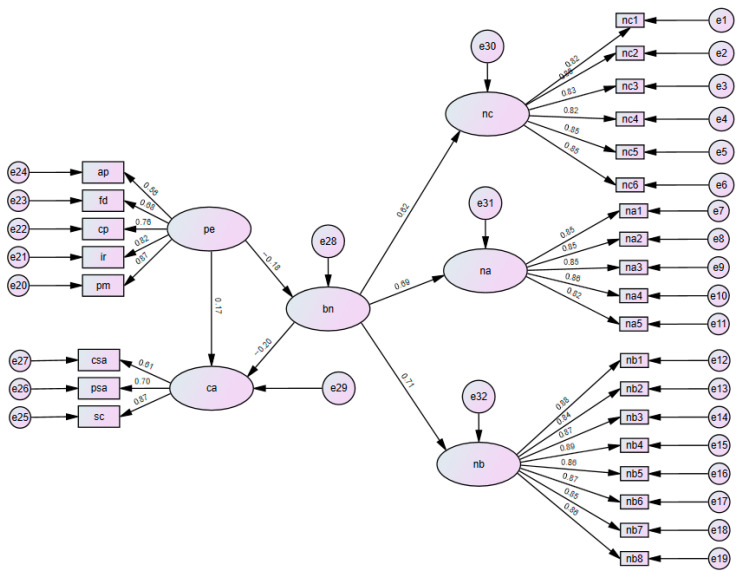
A Standardized Structural Equation Model of Parental Expectations, Basic Psychological Needs Satisfaction, and Competitive State Anxiety. Note. The model controls for gender, age, training experience, and SES (covariates not shown for visual clarity). Ovals represent latent constructs; rectangles represent observed variables. pe = Parental Expectations; bn = Basic Psychological Needs Satisfaction (second-order construct comprising nc = Competence, na = Autonomy, nb = Relatedness); ca = Competitive State Anxiety. Path coefficients are standardized estimates. All solid paths are significant at *p* < 0.05.

A Structural Equation Model (SEM) was examined using Maximum Likelihood estimation. As visualized in Figure 3, the measurement model demonstrated robust psychometric properties, with factor loadings ranging from 0.56 to 0.89 (*p* < 0.001). The structural model yielded an excellent fit to the data ($\chi^{2}/df=1.022$, CFI = 0.999, TLI = 0.999, RMSEA = 0.007).

Table 3 presents the decomposition of effects derived from bias-corrected bootstrapping (5000 resamples).

The total effect of Parental Expectations on Competitive State Anxiety was significant (β = 0.124, *p* < 0.01). The results supported a partial mediation model: Parental Expectations exerted a significant direct effect on anxiety (Direct Effect = 0.111, *p* < 0.01, 95% CI [0.038, 0.184]) and a significant indirect effect via Basic Psychological Needs Satisfaction (Indirect Effect = 0.013, *p* < 0.001, 95% CI [0.007, 0.032]).

To assess specific pathways, a parallel multiple mediation analysis was conducted (Table 3, lower section). The indirect effect was transmitted significantly through the Need for Competence (Indirect Effect = 0.019, *p* < 0.001, 95% CI [0.003, 0.044]). In contrast, the indirect pathways via the Need for Autonomy (Indirect Effect = 0.013, 95% CI [−0.002, 0.035]) and the Need for Relatedness (Indirect Effect = 0.005, 95% CI [−0.027, 0.015]) were not statistically significant.

## 4. Discussion

This study elucidates the mechanism by which parental expectations influence competitive state anxiety through the lens of Self-Determination Theory (SDT) [32,33]. By employing a mediation analysis that controlled for demographic and background variables, we found that parental expectations function as a double-edged sword: they directly predict higher competitive state anxiety and indirectly contribute to it by compromising basic psychological needs satisfaction—most notably, the need for competence [34,35]. These results extend the application of SDT to junior competitive tennis and offer critical insights for managing pre-competition anxiety [36].

### 4.1. The Direct Effect of Parental Expectations on Competitive State Anxiety

The present study revealed a significant positive association between parental expectations and competitive state anxiety among adolescent tennis players (B = 0.111, *p* < 0.01). Our correlation analysis further indicated high internal consistency across the five dimensions of parental expectations. This suggests that parental pressure tends to be generalized; parents who enforce high standards in one domain likely extend them to others, forming a comprehensive system of external pressure that leaves the athlete with little psychological respite. This aligns with literature suggesting that excessive parental involvement often manifests as a critical external stressor in youth sports [37,38].

Consistent with Multidimensional Anxiety Theory [39], high expectations may exacerbate cognitive anxiety by heightening concerns regarding performance evaluation. The strong inter-correlations observed between cognitive and somatic anxiety in our preliminary analysis confirm that psychological distress and physiological arousal, while distinct, are closely linked components that collectively undermine athlete confidence. In junior tennis, an individual sport characterized by isolated performance accountability, athletes are particularly susceptible to this compounded pressure. When expectations exceed perceived coping capabilities, they trigger a “fear of failure,” a primary antecedent of competitive anxiety [40,41]. Furthermore, high expectations are often perceived as parental conditional regard, where affection is contingent upon success, thereby intensifying psychological distress [42,43]. From an SDT perspective, such controlling environments create internal conflict and “introjected” regulation, directly fueling anxiety [14,44,45].

### 4.2. The Mediating Role of Basic Psychological Needs Satisfaction

The mediation analysis elucidated a critical pathway: parental expectations indirectly influence anxiety by compromising competence satisfaction (β = −0.18, *p* = 0.005), which in turn predicts elevated anxiety (β = −0.20, *p* = 0.004). The observed negative correlations between parental expectations and basic needs in our results corroborate the theoretical notion that heightened external expectations function as a “controlling environmental factor.”

These findings support Basic Psychological Needs Theory [14,20,46]. When parents impose unrealistically high standards, they inadvertently create a “perceived discrepancy” between the athlete’s current ability and the expected outcome. This discrepancy fosters self-doubt and diminishes perceived efficacy. This link aligns with the Control-Value Theory [47,48,49]: anxiety is triggered when an individual values an outcome but perceives a lack of control over achieving it. Consequently, a compromised sense of competence leaves athletes vulnerable to pre-competitive distress [20,32].

### 4.3. The Unique Mediating Role of the Need for Competence

Notably, only the need for competence significantly mediated the relationship between parental expectations and anxiety (B = 0.019, *p* < 0.001), while autonomy and relatedness did not. This underscores the context-specific nature of competitive sports.

In achievement settings like tennis, individuals are primarily driven by mastery [21]. Parental expectations focus on rankings and match results, mapping directly onto the athlete’s self-evaluation of ability. Research specifically in racket sports indicates that the solitary nature of the game amplifies the reliance on self-efficacy for emotional regulation [50]. Thus, the immediate psychological threat of parental pressure is a challenge to perceived efficacy rather than volition or belonging. Since anxiety is fundamentally a reaction to a perceived inability to meet demands, competence satisfaction serves as the most proximal predictor [51,52].

### 4.4. The Dual Pathways of Parental Expectations: Direct and Indirect Effects

The findings substantiate a partial mediation model, suggesting a dual-process mechanism. Parental expectations operate concomitantly as a disruptor of internal resources (via compromised competence) and as a salient external stressor. This divergent relationship was visually corroborated by our heat map analysis (Figure 2), where the distinct clustering of positive associations (between expectations and anxiety) contrasted sharply with the protective, inverse relationships involving need satisfaction.

The significant direct effect implies that high parental expectations likely function as a social evaluative threat beyond SDT’s scope. Drawing upon the Transactional Theory of Stress [53,54], athletes may appraise high expectations as a demanding constraint exceeding their coping resources. This highlights that anxiety is driven not only by self-doubt (indirect path) but also by the cognitive appraisal of potential consequences, such as parental disappointment [55,56].

### 4.5. Contextual and Cultural Interpretations of the Null Findings

The non-significant indirect effects of autonomy and relatedness warrant a cautious interpretation regarding domain specificity and culture.

First, competitive junior tennis operates within an outcome-oriented framework where self-esteem is often tethered to achievement [57,58]. The most salient threat is likely a deficit in skill (competence) rather than social support.

Second, cultural factors may provide a plausible explanation for these findings. In the Chinese context, grounded in Confucian heritage, fulfilling parental expectations is often internalized as a moral obligation (filial piety) rather than purely external control [59,60]. Consequently, Chinese junior athletes may not interpret high expectations as a violation of autonomy. However, the magnitude of these expectations still imposes a burden on perceived competence. We hypothesize that in collectivist cultures, the psychological cost of parental pressure manifests more acutely through the erosion of confidence rather than the suppression of will [61].

## 5. Limitations, Future Research, and Practical Implications

### 5.1. Limitations and Future Research Directions

Despite the theoretical and empirical contributions of this study, several limitations warrant consideration. First, the cross-sectional design precludes causal inferences regarding the temporal ordering of parental expectations, need satisfaction, and state anxiety. Future research should employ longitudinal or cross-lagged panel designs to rigorously establish causality and examine the dynamic interplay of these variables over a competitive season.

Second, this study relied exclusively on self-report measures from adolescent athletes, which may introduce common method variance and subjective bias. Although statistical tests indicated minimal bias, future studies would benefit from a multi-informant approach, incorporating objective data (e.g., physiological markers of anxiety) or dyadic data matched with parents’ self-reported expectations to capture a more holistic view of the parent–child dynamic.

Third, the sample was restricted to Chinese junior tennis players, a group influenced by specific cultural values (e.g., filial piety) and the unique pressures of an individual sport. Our finding—that competence, but not autonomy or relatedness, mediated the anxiety response—may be context-specific. Future investigations should test the generalizability of this model across different cultural backgrounds (e.g., Individualistic vs. Collectivist) and sport types (e.g., Team vs. Individual sports).

### 5.2. Practical Implications

The findings provide critical insights for stakeholders in youth sports. To address the “double-edged” nature of high expectations, sports organizations should implement parent education workshops. These sessions aim to help parents distinguish between supportive involvement and controlling pressure, encouraging a shift from “outcome-oriented” demands to “process-oriented” support. By reducing the emphasis on external validation, parents can help preserve the athlete’s self-efficacy.

Furthermore, given that the need for competence was the sole mediator, coaches play a vital role in buffering against parental pressure. Beyond technical training, coaches should strive to build an autonomy-supportive sport environment. Strategies such as involving athletes in goal-setting and providing choices during practice can enhance the athlete’s sense of ownership. Additionally, creating a mastery-motivational climate—where success is defined by personal improvement rather than ranking—can directly bolster competence satisfaction, thereby alleviating pre-competitive anxiety.

## 6. Conclusions

Situated within the context of junior competitive tennis, this study clarifies the mechanism linking parental expectations to athlete well-being. The results substantiate a “dual-pathway” model: parental expectations exert a robust direct effect on competitive state anxiety while simultaneously exerting an indirect effect by specifically undermining the need for competence. Unlike general theoretical assumptions, the needs for autonomy and relatedness did not mediate this relationship, suggesting that in this achievement-focused individual sport, anxiety is primarily driven by a crisis of confidence (“Can I meet the standard?”) rather than a loss of volition. Collectively, these findings highlight that alongside managing external parental pressure, protecting and nurturing a young athlete’s sense of competence is the most critical internal defense against pre-competitive distress.

## Figures and Tables

**Table 1:** The Reliability and Validity Indices of the Study Variables.

Variable	Cronbach’s α (Total)	Factors	Items	Cronbach’s α (Subscale)
Parental Expectations	0.954	Academic Performance	4	0.867
		Future Development	4	0.902
		Interpersonal Relations	6	0.844
		Conduct	3	0.929
		Physical/Mental Quality	7	0.941
Basic Psychological Needs	0.949	Competence	6	0.937
		Autonomy	5	0.931
		Relatedness	8	0.962
Competitive State Anxiety	0.933	Cognitive Anxiety	9	0.860
		Somatic Anxiety	9	0.865
		Self-Confidence	9	0.822

**Table 2:** Correlation Analysis of Variables Between Competitive State Anxiety and Parental Expectations.

Variables	1	2	3	4	5	6	7	8	9	10	11
Academic Performance	--										
Future Development	0.348 **	--									
Interpersonal Relations	0.371 **	0.518 **	--								
Conduct	0.488 **	0.563 **	0.629 **	--							
Physical/Mental Quality	0.447 **	0.594 **	0.648 **	0.705 **	--						
Competence Needs	−0.105	−0.163 *	−0.240 **	−0.145 *	−0.239 **	--					
Autonomy Needs	0.038	−0.131 *	−0.079	−0.027	−0.131 *	0.435 **	--				
Relatedness Needs	−0.030	−0.078	−0.113	−0.095	−0.181 **	0.479 **	0.547 **	--			
Cognitive State Anxiety	0.145 *	0.095	0.170 **	0.062	0.102	−0.177 **	−0.246 **	−0.141 *	--		
Somatic State Anxiety	0.109	0.060	0.176 **	0.054	0.063	−0.168 **	−0.194 **	−0.133 *	0.862 **	--	
State Self-Confidence	0.124	0.132 *	0.175 **	0.115	0.180 **	−0.190 **	−0.278 **	−0.195 **	0.611 **	0.604 **	--
M (Mean)	3.01	3.05	3.04	3.06	3.10	4.18	4.15	4.15	1.94	1.91	1.82
SD (Standard Deviation)	1.05	1.09	1.11	1.15	1.10	1.42	1.47	1.56	0.55	0.57	0.49

Note: * *p* < 0.05, ** *p* < 0.01.

**Table 3:** The Path Coefficients of the Relationship Between Competitive State Anxiety and Parental Expectations.

Effect	Path	Estimate	95%CI	
			LL	UL
Total Effect	pe→bn→ca	0.124 **	0.051	0.197
Indirect Effect		0.013 ***	0.0007	0.032
Direct Effect		0.111 **	0.038	0.184
Total Effect	pe→nc→ca	0.124 **	0.051	0.197
Indirect Effect		0.019 ***	0.003	0.044
Direct Effect		0.110 **	0.036	0.183
Total Effect	pe→na→ca	0.124 **	0.051	0.197
Indirect Effect		0.013	−0.002	0.035
Direct Effect		0.115 **	0.042	0.187
Total Effect	pe→nb→ca	0.124 **	0.051	0.197
Indirect Effect		0.005	−0.027	0.015
Direct Effect		0.120 **	0.047	0.193

Note: ** *p* < 0.01, *** *p* < 0.001.

## Data Availability

The data presented in this study are available on request from the corresponding author.
